# Impact and clinical profiles of *Mycoplasma pneumoniae* co-detection in childhood community-acquired pneumonia

**DOI:** 10.1186/s12879-019-4426-0

**Published:** 2019-10-11

**Authors:** Meng-chuan Zhao, Le Wang, Fang-zhou Qiu, Li Zhao, Wei-wei Guo, Shuo Yang, Zhi-shan Feng, Gui-xia Li

**Affiliations:** 1 Institute of Pediatric Research, Children’s Hospital of Hebei Province, affiliated to Hebei Medical University, 133 Jianhua South Street, Shijiazhuang, 050031 Hebei Province China; 2 Graduate School of Hebei Medical University, 361 Zhongshan East Road, Shijiazhuang, 050017 Hebei Province China; 3 Department of Laboratory Medicine, People’s Hospital of Hebei Province, 384 Heping West Road, Shijiazhuang, 050051 Hebei Province China

**Keywords:** Co-detection, *Mycoplasma pneumoniae*, Outcomes, Community-acquired pneumonia, Children

## Abstract

**Background:**

Increasing number of hospitalized children with community acquired pneumonia (CAP) is co-detected with *Mycoplasma pneumoniae* (*Mp*). The clinical characteristics and impact of *Mp* co-detected with other bacterial and/or viral pathogens remain poorly understood. The purpose of this study was to evaluate the demographic and clinical features of CAP children with *Mp* mono-detection and *Mp* co-detection.

**Methods:**

A total of 4148 hospitalized children with CAP were recruited from January to December 2017 at the Children’s Hospital of Hebei Province, affiliated to Hebei Medical University. A variety of respiratory viruses, bacteria and *Mp* were detected using multiple modalities. The demographic and clinical features of CAP children with *Mp* mono-detection and *Mp* co-detection were recorded and analyzed.

**Results:**

Among the 110 CAP children with *Mp* positive, 42 (38.18%) of them were co-detected with at least one other pathogen. Co-detection was more common among children aged ≤3 years. No significant differences were found in most clinical symptoms, complications, underlying conditions and disease severity parameters among various etiological groups, with the following exceptions. First, prolonged duration of fever, lack of appetite and runny nose were more prevalent among CAP children with *Mp*-virus co-detection. Second, *Mp*-virus (excluding *HRV*) co-detected patients were more likely to present with prolonged duration of fever. Third, patients co-detected with *Mp-*bacteria were more likely to have abnormal blood gases. Additionally, CAP children with *Mp*-*HRV* co-detection were significantly more likely to report severe runny nose compared to those with *Mp* mono-detection.

**Conclusion:**

*Mp* co-detection with viral and/or bacterial pathogens is common in clinical practice. However, there are no apparent differences between *Mp* mono-detection and *Mp* co-detections in terms of clinical features and disease severity.

## Background

Community-acquired pneumonia (CAP) is a leading cause of hospitalization among infants and children worldwide, especially in developing countries. A variety of respiratory viruses such as *Influenza A* (*Flu A*), *respiratory syncytial virus* (*RSV*), *adenovirus* (*ADV*) and *human metapneumovirus* (*HMPV*), and bacteria such as *Streptococcus pneumoniae*, *Hemophilus influenza*, *Staphylococcus aureus* and *Mycoplasma pneumoniae* (*Mp*) are associated with childhood CAP [1–4]. Notably, *Mp* accounts for 10–40% of CAP in children, and as high as 18% of cases with CAP require hospitalization [4–6]. In recent years, with the development more frequent use of molecular diagnostics, an increasing number of hospitalized CAP children are diagnosed with mixed viral-bacterial detections, and *Mp* co-detection is not uncommon [7–10]. However, the clinical characteristics and implications of *Mp* co-detection are poorly described.

To better characterize the impacts of *Mp* co-detection on CAP children, a 12-month prospective study was carried out to examine the demographic and clinical characteristics of CAP caused by *Mp*, including mono-detection and co-detection. In addition, this study assessed the differences in demographic and clinical features between mono- and co-detected CAP children.

## Methods

### Study population and case definitions

This prospective study was conducted on children (< 18 years) with CAP admitted at the Children’s Hospital of Hebei Province, affiliated to Hebei Medical University, China, over a period of 12 months (from January 1, 2017 to December 31, 2017). The diagnostic criteria for CAP included: (a) clinical manifestations: fever, cough and/or dyspnea; (b) auscultatory findings: abnormal breath sounds, wheezes or crackles; and (c) radiographic evidence: consolidation, other infiltrate or pleural effusion.

CAP patients with positive results of *Mp-DNA* bronchoalveolar lavage fluid (BALF) or a ≥ 4-fold rise in IgG titer were considered to have *Mp* positive. If only *Mp* was detected, the patients were considered to have *Mp* mono-detection. Co-detection was defined as detection of *Mp* with ≥1 other bacterial or viral pathogen. Children with co-detection were stratified into two groups: *Mp*-virus co-detection and *Mp-*bacteria co-detection. The *Mp*-virus co-detection group was further stratified into two subgroups: *Mp*-*HRV* co-detection and *Mp*-virus (excluding *HRV*) co-detection.

### Data collection

The demographic, clinical, laboratory and other related data of *Mp-*positive children were collected: (1) demographic characteristics: gender and age; (2) clinical information: duration of fever, cough, wheezing and gastrointestinal symptoms (diarrhea or vomiting); (3) laboratory results: peripheral leukocyte, neutrophil and lymphocyte counts, C-reactive protein (CRP) levels and chest radiographic findings; and (4) disease severity parameters: incidence of severe CAP [11], length of hospitalization, requirement for mechanical ventilation and admission to the PICU.

### Specimen collection and laboratory testing

Blood, serum, nasopharyngeal aspirates (NPAs) and BALF specimens were obtained for pathogen detection using multiple modalities.

Real-time polymerase chain reaction (PCR) was performed for *Mp-DNA* detection in BALF specimens using a quantitative diagnostic kit (DaAn Gene Co. Ltd., Guangzhou, China) [12]. Serum IgG against *Mp* was evaluated by a commercial test kit (Virion-Serion, Germany) according to the manufacturer’s instructions. Criteria for the diagnostic test were defined as IgG ≥4-fold titers [13].

The NPAs collected from all patients were tested simultaneously for *Flu A, Influenza B* (*Flu B*), *Influenza A H1N1 pdm09* (*09H1*), *influenza H3N2* (*H3*), *human parainfluenza virus* (*HPIV*), *RSV*, *rhinovirus* (*HRV*), *ADV*, *HMPV*, *human bocavirus* (*HBoV*), *human coronavirus* (*HCoV*), *Chlamydia* (*Ch*) and *Mp* using a GeXP-based multiplex reverse transcription PCR assay [14]. The presence of bacteria and fungi was determined on admission by a positive result in blood specimen and/or BALF culture with the use of standard techniques.

### Statistical analysis

Statistical analyses were performed using SPSS 13.0.1 statistics package (SPSS Inc., Chicago, USA). In brief, the demographic and clinical manifestations of all children with *Mp* positive were presented as absolute frequencies or rates for categorical variables, median (interquartile range) values for quantitative variables. Multiple sets of independent continuous data were compared using the Kruskale-Wallis test, while two sets of independent continuous data were compared by the Mann-Whitney U test. Categorical variables were analyzed using the Pearson Chi-square or Fisher’s Exact Test. The level of statistical significance was set at *P* < 0.05.

## Results

### Study population and microbiological diagnosis

Between January 1, 2017 and December 31, 2017, 4148 patients who met the criteria of CAP were enrolled. Among them, 110 (2.65%) were defined as *Mp* positive (Table 1). Of these 110 patients, 68 (61.82%) were detected with only *Mp*, 42 (38.18%) were detected with at least one other pathogen, 30 (27.27%) were detected with one or more viruses, 7 (6.36%) were detected with one or more bacteria, and the remaining 5 (4.55%) were detected with both bacterial and viral pathogens (Table 2).

**Table 1:** The results of *Mp* detection for 4148 patients with CAP

Results	*Mp* -DNA (BALF)	*Mp* -DNA (NPA)^a^	IgG(paired sera)	NO.
*Mp* positive (*N* = 110)	+	+	/	98
	+	+	+	6
	+	+	/	6
*Mp* negative (*N* = 4038)	–	+	/	4
	/	+	/	62
	/	–	/	3941
	/	–	–	31

*Mp Mycoplasma pneumoniae*, *CAP* community-acquired pneumonia, *BALF* bronchoalveolar lavage fluid, *NPAs* nasopharyngeal aspirates

^a^The NPAs were tested simultaneously for *Mp*, *Chlamydia* and 11 common respiratory viruses using a GeXP-based multiplex reverse transcription PCR assay, thus *Mp*-DNA (NPAs) was tested for all patients

**Table 2:** Etiologic agents of 110 children with *Mp* positive

Etiologic agents	Proportion (%)
*Mp* mono-detection	68(61.82)
*Mp* co-detection	42(38.18)
Virus	30(27.27)
*Mp* + *HRV*	20
*Mp* + *HPIV*	6
*Mp* + *HRV* + *HPIV*	1
*Mp* + *ADV*	1
*Mp* + *HCoV*	1
*Mp* + *H3*	1
Bacteria	7(6.36)
*Mp* + *S. pneumoniae*	1
*Mp* + *S. epidermidis*	1
*Mp* + *Alcaligenes spp*	1
*Mp* + *A. baumannii*	1
*Mp* + *Radioactive rhizobia*	1
*Mp* + *S. viridans*	1
*Mp* + *S. saprophyticus*	1
Bacteria and virus	5(4.55)
*Mp* + *HRV* + *S. pneumoniae*	1
*Mp* + *HMPV* + *S. marcescens*	1
*Mp* + *ADV* + *S. hominis*	1
*Mp* + *HBoV* + *K. oxytoca*	1
*Mp* + *HMPV* + *HRV* + *A. baumannii*	1

*Mp Mycoplasma pneumoniae*, *HRV Rhinovirus*, *RSV Respiratory syncytial virus*, *ADV Adenovirus*, *HPIV Human parainfluenza virus*, *HMPV Human metapneumovirus*, *H3 Influenza H3N2*, *HCoV Human coronavirus*, *HBoV Human bocavirus*, *S. pneumoniae Streptococcus pneumoniae*, *S. epidermidis Staphylococcus epidermidis*, *S. marcescens Serratia marcescens*, *A. baumannii Acinetobacter baumannii*, *S. viridans Streptococcus viridans*, *S. saprophyticus Staphylococcus saprophyticus, S. hominis Staphylococcus hominis*, *K. oxytoca Klebsiella oxytoca*

### Demographic characteristics

**Fig. 1:**
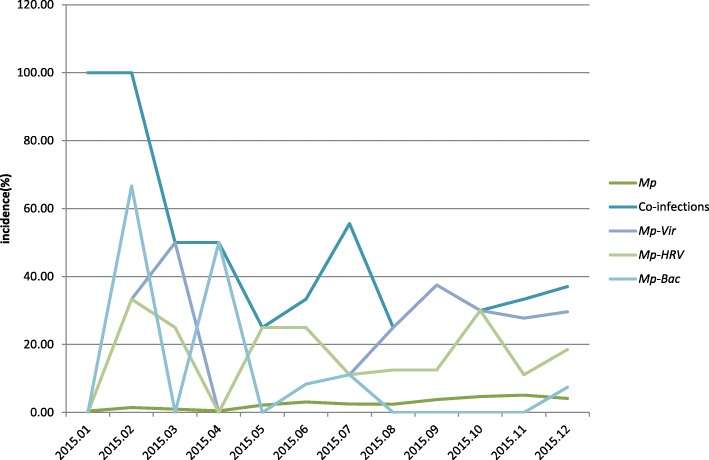
Seasonal distribution of *Mp* and *Mp* co-detection. *Mp*: *Mycoplasma pneumoniae*, *Mp*-Vir: *Mp*-virus co-detection, *Mp*-*HRV*: *Mp*-*HRV* co-detection, *Mp*-Bac: *M*p-bacteria co-detection

Among the 110 patients with *Mp* positive, the median age was 5 years, which was significantly higher compared to that of 4148 CAP children (median age: 0.5 years, *P* < 0.001). Approximately 57.3% were male, and the gender distribution did not differ significantly between children with *Mp* positive and CAP (*P* = 0.094). Co-detection was more prevalent in children aged ≤3 year compared to those aged > 3 years old (64.29% vs. 29.27%; *P* = 0.001). The median age of CAP children with *Mp*-virus co-detection (*P* = 0.009), *Mp*-*HRV* co-detection (*P* = 0.004) and *Mp*-virus (excluding *HRV*) co-detection (*P* = 0.045) was significantly younger compared to those with *Mp* mono-detection (Table 3). Gender distribution did not differ significantly among various etiological groups (Table 3). The peak incidence of *Mp* detection was in autumn (October to November), while the peak incidence of *Mp* co-detection was in winter. Besides, *Mp*-virus co-detection and *Mp-HRV* co-detection occurred throughout the year, with no obvious seasonality. For *Mp-*bacteria co-detection, the highest incidence rates were observed in winter and spring (Fig. 1).

**Table 3:** The demographic characteristics among various etiological groups

Characteristics	*Mp* (*n* = 68)	*Mp*-Vir (*n* = 30)	*P*	*Mp-HRV* (*n* = 20)	*P*	*Mp*-Vir (excluding *HRV*) (*n* = 10)	*P*	*Mp*-Bac (*n* = 7)	*P*
Age, median (IQR) (y)	6 (3)	4 (3)	0.009	4 (3)	0.004	5 (3.5)	0.045	5 (5)	0.514
≤1 year	0 (0)	1 (3.33)	0.02	0 (0)	0.011	1 (10)	0.205	0 (0)	0.52
1 to ≤3 years	10 (14.71)	10 (33.33)		9 (45)		1 (10)		2 (28.57)	
3 to ≤6 years	30 (44.12)	13 (43.33)		8 (40)		5 (50)		3 (42.86)	
> 6 years	28 (41.18)	6 (20)		3 (15)		3 (30)		2 (28.57)	
Sex, male	37 (54.41)	19 (63.33)	0.411	13 (65)	0.401	6 (60)	1	4 (57.14)	1
Seasons									
Summer(6–8)	18 (26.47)	6 (20)	0.929	5 (25)	0.966	1 (10)	0.705	2 (28.57)	0.107
Autumn(9–11)	24 (35.29)	11 (36.67)		6 (30)		5 (50)		0 (0)	
Winter(12–2)	17 (25)	9 (30)		6 (30)		3 (30)		4 (57.14)	
Spring(3–5)	9 (13.24)	4 (13.33)		3 (15)		1 (10)		1 (14.29)	

Data are presented as No. (%) unless otherwise indicated

*Mp Mycoplasma pneumoniae*, *Mp*-Vir *Mp*-virus co-detection, *Mp*-*HRV Mp*-*HRV* co-detection, *Mp*-Bac *Mp*-bacteria co-detection, *IQR* interquartile range

### Clinical data, laboratory and radiographic findings

The differences in clinical data, laboratory and radiographic findings among various etiological groups are summarized in Table 4. Fever, cough, and sore throat were the three most common symptoms of children with *Mp* positive. In comparison with mono-detected patients, the duration of fever was significantly longer in *Mp*-virus co-detected (*P* = 0.039) and *Mp*-virus (excluding *HRV*) co-detected (*P* = 0.031) patients. Lack of appetite was more prevalent among children with *Mp*-virus co-detection (*P* = 0.022). Runny nose was more common in patients with *Mp*-virus co-detection (*P* = 0.012) and *Mp*-*HRV* co-detection (*P* = 0.002). However, the clinical features of patients with *Mp-*bacteria co-detection were relatively similar to those with *Mp* mono-detection. No significant differences were noted in terms of the laboratory and radiographic findings among various etiological groups.

**Table 4:** Clinical data, laboratory and radiographic findings among various etiological groups

Characteristics	*Mp* (*n* = 68)	*Mp*-Vir (*n* = 30)	*P*	*Mp-HRV* (*n* = 20)	*P*	*Mp*-Vir (excluding *HRV*) (*n* = 10)	*P*	*Mp*-Bac (*n* = 7)	*P*
Clinical data									
Fever	68 (100)	29 (96.67)	0.306	20 (100)	–	9 (90)	0.128	7 (100)	–
Days of total, median(IQR) (d)	12 (7)	14 (6)	0.039	13.5 (8.75)	0.212	16 (4.5)	0.031	11 (7)	0.935
Cough	68 (100)	28 (93.33)	0.092	18 (90)	0.05	10 (100)	–	7 (100)	–
Fatigue	6 (8.82)	6 (20)	0.222	4 (20)	0.325	2 (20)	0.596	1 (14.29)	0.512
Lack of appetite	16 (23.53)	14 (46.67)	0.022	9 (45)	0.061	5 (50)	0.168	2 (28.57)	1
Dyspnea	7 (10.29)	4 (13.33)	0.927	2 (10)	1	2 (20)	0.714	1 (14.29)	0.562
Chills	4 (5.88)	3 (10)	0.761	2 (10)	0.891	1 (10)	0.506	1 (14.29)	0.396
Headache	3 (4.41)	1 (3.33)	1	1 (5)	1	0 (0)	1	0 (0)	1
Sore throat	66 (97.06)	30 (100)	1	20 (100)	1	10 (100)	1	7 (100)	1
Wheezing	1 (1.47)	3 (10)	0.158	2 (10)	0.128	1 (10)	0.241	0 (0)	1
Runny nose	0 (0)	4 (13.33)	0.012	4 (20)	0.002	0 (0)	–	0 (0)	–
Abdominal pain	3 (4.41)	1 (3.33)	1	1 (5)	1	0 (0)	1	1 (14.29)	0.33
Diarrhea	1 (1.47)	0 (0)	1	0 (0)	1	0 (0)	1	0 (0)	1
Vomit	3 (4.41)	3 (10)	0.544	2 (10)	0.689	1 (10)	0.429	0 (0)	1
Chest pain	5 (7.35)	0 (0)	0.305	0 (0)	0.484	0 (0)	1	0 (0)	1
Rash	2 (2.94)	1 (3.33)	1	1 (5)	0.543	0 (0)	1	0 (0)	1
Laboratory findings									
Leukocyte count, median(IQR) × 10^9^/L	10.35 (5.85)	10.4 (5.8)	0.691	10.4 (4.55)	0.812	11 (12.2)	0.639	12.4 (7.1)	0.303
Neutrophils, median(IQR) (%)	64.55 (13.58)	59.9 (18.6)	0.137	60.6 (18.25)	0.142	58.55 (27.33)	0.509	60 (16.5)	0.549
Lymphocytes, median (IQR) (%)	25.45 (12.03)	27.5 (15.1)	0.244	27.5 (19.45)	0.133	23.45 (19.23)	0.872	27.3 (17.1)	0.888
C-reactive protein, median(IQR)(mg/L)	20.6 (36.55)	9.7 (34.45)	0.063	9.3 (34.3)	0.06	16.85 (58.15)	0.459	8.7 (23)	0.441
Radiographic findings									
Single lobar infiltrate	27 (39.71)	12 (40)	0.978	10 (50)	0.412	2 (20)	0.393	4 (57.14)	0.625
Multilobar infiltrates (unilateral)	19 (27.94)	6 (20)	0.406	3 (15)	0.24	3 (30)	1	0 (0)	0.245
Multilobar infiltrates (bilateral)	19 (27.94)	12 (40)	0.237	7 (35)	0.543	5 (50)	0.296	3 (42.86)	0.697
Bronchitis	3 (4.41)	0 (0)	0.551	0 (0)	1	0 (0)	1	0 (0)	1
Hilar lymphadenopathy	2 (2.94)	3 (10)	0.334	3 (15)	0.134	0 (0)	1	0 (0)	1

Data are presented as No. (%) unless otherwise indicated

*Mp Mycoplasma pneumoniae*, *Mp*-Vir *Mp*-virus co-detection, *Mp*-*HRV Mp*-*HRV* co-detection, *Mp*-Bac *Mp*-bacteria co-detection, *IQR* interquartile range

### Complications, underlying conditions and disease severity parameters

The differences in complications, underlying conditions and disease severity parameters among various etiological groups are presented in Table 5. All children with *Mp* positive, including *Mp* mono-detection and *Mp* co-detection, did not require PICU admission or invasive mechanical ventilation. However, the cases with severe CAP might require noninvasive mechanical ventilation, and the duration of hospitalization was not significantly different between the patients with *Mp* co-detection and *Mp* mono-detection. However, compared to those with *Mp* mono-detection, *Mp-*bacteria co-detected children were more likely to have abnormal blood gases (*P* = 0.041). In addition, the underlying medical conditions of *Mp* co-detected patients were relatively similar to those of *Mp* mono-detected patients.

**Table 5:** Complications, underlying conditions and disease severity parameters among various etiological groups

Characteristic	*MP* (*n* = 68)	*Mp*-Vir (*n* = 30)	*P*	*Mp*-*HRV* (*n* = 20)	*P*	*Mp*-Vir (excluding *HRV*) (*n* = 10)	*P*	*Mp*-Bac (*n* = 7)	*P*
Complications^a^									
Any condition (≥1 condition)	55 (80.88)	29 (96.67)	0.081	20 (100)	0.078	9 (90)	0.795	7 (100)	0.454
Pleural effusion	35 (51.47)	13 (43.33)	0.458	6 (30)	0.091	7 (70)	0.449	5 (71.43)	0.542
Small	1 (1.47)	1 (3.33)	0.144	1 (5)	0.092	0 (0)	0.433	2 (28.57)	0.073
Moderate	33 (48.53)	10 (33.33)		4 (20)		6 (60)		3 (42.86)	
Massive	1 (1.47)	2 (6.67)		1 (5)		1 (10)		0 (0)	
Consolidation	37 (54.41)	20 (66.67)	0.257	13 (65)	0.401	7 (70)	0.557	7 (100)	0.054
Lobar or unilateral change	34 (50)	17 (56.67)	0.721	11 (55)	0.83	6 (60)	0.513	6 (85.71)	0.513
Bilateral pulmonary multiple change	3 (4.41)	3 (10)		2 (10)		1 (10)		1 (14.29)	
Pulmonary atelectasis	7 (10.29)	5 (16.67)	0.581	5 (25)	0.189	0 (0)	0.586	2 (28.57)	0.196
Liver dysfunction	8 (11.76)	4 (13.33)	1	3 (15)	1	1 (10)	1	0 (0)	1
Myocardial damage	18 (26.47)	7 (23.33)	0.743	5 (25)	0.895	2 (20)	0.96	1 (14.29)	0.803
Abnormal blood gas (hypoxemia, hypokalemia, etc.)	2 (2.94)	1 (3.33)	1	0 (0)	1	1 (10)	0.341	2 (28.57)	0.041
Central nervous system infection	1 (1.47)	1 (3.33)	0.521	0 (0)	1	1 (10)	0.241	0 (0)	1
Underlying conditions									
Any condition (≥1 condition)	8 (11.76)	4 (13.33)	1	3 (15)	1	1 (10)	1	1 (14.29)	1
Respiratory disease (asthma, congenital malformation etc.)	2 (2.94)	2 (6.67)	0.76	1 (5)	0.543	1 (10)	0.341	0 (0)	1
Neurological Disease (epilepsy)	2 (2.94)	0 (0)	1	0 (0)	1	0 (0)	1	0 (0)	1
Congenital heart disease	0 (0)	2 (6.67)	0.092	2 (10)	0.05	0 (0)	–	0 (0)	–
Disease severity parameters									
Severe CAP^b^	49 (72.06)	19 (63.33)	0.388	10 (50)	0.065	9 (90)	0.409	6 (85.71)	0.742
PICU admission	0 (0)	0 (0)	–	0 (0)	–	0 (0)	–	0 (0)	–
Noninvasive positive pressure ventilation	4 (5.88)	2 (6.67)	1	1 (5)	1	1 (10)	0.506	0 (0)	1
Invasive mechanical ventilation	0 (0)	0 (0)	–	0 (0)	–	0 (0)	–	0 (0)	–
Length of stay, median(IQR) (d)	13 (5)	14 (6)	0.177	14.5 (6)	0.163	13.5 (7)	0.599	11 (3)	0.319

Data are presented as No. (%) unless otherwise indicated

*Mp Mycoplasma pneumoniae*, *Mp*-Vir *Mp*-virus co-detection, *Mp*-*HRV Mp*-*HRV* co-detection, *Mp*-Bac *Mp*-bacteria co-detection, *IQR* interquartile range

^a^All cases of pulmonary atelectasis are single lobar; All cases of liver dysfunction, myocardial damage and abnormal blood gas are mild;

^b^The diagnosis criteria of severe CAP are in accordance with the Chinese Medical Association Guidelines for the management of community-acquired pneumonia in children (revised 2013) (Chin J Pediatr. 2013; 51(11):856–62)

## Discussion

*Mp* has been recognized as a significant and common cause of pediatric CAP. In this study, it was found that 2.65% of hospitalized children with CAP experienced *Mp* positive, which is less than 19.1% reported in Wuhan, China [15]. Such low incidence may be attributed to the different study populations and methods used for *Mp* testing. Previous studies [16–18] have indicated that *Mp* is detected year-round, and its seasonal peaks have been reported in various seasonal periods starting from the end of summer to winter. In this study, *Mp* positive was more common in autumn, which is consistent with the findings of Chiu et al. [19].

At present, it is widely considered that mixed detections with multiple pathogens are common in children with CAP. Furthermore, *Mp* infection is often associated with preceding or concomitant viral and bacterial infections in children [19, 20]. In the present study, we found that children with co-detection accounted for 38.18% of total cases with *Mp* positive. This proportion is lower than that in Taiwan population [19] and higher than that in Beijing population [20]. However, the incidence of mixed detections with viruses was 27.27% in this study, which is higher compared to both Taiwan and Beijing populations. These differences may be due to the exclusion of *HRV* test in their studies. *HRV* is the most prevalent respiratory virus and is often associated with the common cold. Moreover, *HRV* may be associated with more severe lower respiratory tract infections in children, including bronchiolitis and pneumonia [21]. The results of this study showed that *HRV* was the most common pathogen among children with *Mp* co-detection, at a frequency of up to 18.18%. Thus, researchers and pediatricians should pay more attention to *Mp*-*HRV* co-detection among children with CAP. Age is an important factor that can affect pathogen distribution. The incidence of *Mp* infection is highest among children aged 3–7 years, while respiratory viral infections are more common in children younger than 2 years [22, 23]. Likewise, in this study, the patients with *Mp*-virus co-detections were significantly younger, especially prevalent among those ≤3 years old.

*Mp-*infected children can present with fever, cough, chest pain and wheeze, along with non-respiratory symptoms such as arthralgia and headache [24]. In the present study, we found that, similar to *Mp* mono-detection, fever, cough and sore throat were the three main symptoms of *Mp* co-detection, followed by non-respiratory symptoms, including rash, headache and abdominal pain. In addition, there were some differences in the clinical symptoms between patients with co-detection and mono-detection. First, compared to *Mp* mono-detection children, the duration of fever was significantly longer in children with *Mp*-virus and *Mp*-virus (excluding *HRV*) co-detection. Second, lack of appetite was more prevalent in children with *Mp*-virus co-detection. Third, runny nose was more common in patients with *Mp*-virus co-detection and *Mp*-*HRV* co-detection. However, the clinical symptoms are relatively non-specific, indicating that these parameters may not be helpful to distinguish *Mp* co-detection from *Mp* mono-detection.

A recent study shows that the clinical outcomes of *Mp* infection are heavily dependent on the co-infected pathogen [20]. Pientong et al. [25] have reported that *Mp* may serve as an important co-infectious agent of respiratory viruses, which increases the severity of acute childhood bronchiolitis. However, no significant differences were noted in the incidence of severe CAP, duration of hospitalization, and noninvasive mechanical ventilation among various etiological groups in the present study, suggesting that mixed detection of *Mp* with other viral or bacterial pathogen does not contribute to the severity of CAP. Chiu et al. [19] demonstrate that no significant difference is observed between the patients infected with *Mp* and those co-infected with virus, as similar to that reported in our study. To avoid overestimation of *Mp*-*HRV* co-detection, we further investigated the differences in clinical outcomes between children with *Mp*-virus (excluding *HRV*) co-detection and *Mp* mono-detection. Similarly, there was no significant difference between the two groups. Then, we further explored the role of *Mp*-*HRV* co-detection in children with CAP. Notably, a similar distribution of most complications, underlying conditions and disease severity parameters were found between the two groups. These results indicate that *Mp-HRV* co-detection may have little influence on the clinical outcomes of CAP. Likewise, we found no significant difference in the clinical outcomes between *Mp-*bacteria co-detection and *Mp* mono-detection, except that *Mp-*bacteria co-detection was more likely to be associated with abnormal blood gases. However, our results might differ from with the findings [19, 20] of different regions of China, suggesting that *S. pneumoniae* co-infection can lead to greater disease severity in children with *Mp* infection compared to single infection.

With the use of molecular diagnostics, co-detection with other viral/bacterial pathogens has been commonly identified in *Mp* positive patients. Nonetheless, this study confirms that the clinical features and severity of *Mp* mono-detected patients are relatively similar to those co-detected with viral and/or bacterial pathogens. Thus, we speculate that a large proportion of CAP patients may be infected with a major pathogen of pneumonia (i.e. *Mp*) and tend to have a colonization of other pathogens in respiratory tract [26]. Furthermore, it is also possible that the viral or bacterial pneumonia patients may have a serologic evidence of past *MP* infection (IgM positive) or PCR positive (colonization).

Nevertheless, this study has several limitations. The presence of co-infection should be confirmed by serologic tests at least 2 times during hospitalization or convalescent stage. However, it is very difficult to apply at clinical fields, owing to lack of available methods. In this study, due to the limits of current respiratory bacteria detection methods, the pattern of identified pathogens might not accurately represent the CAP, especially in young children. Moreover, the small sample size could impose restrictions on determining the association between *Mp* co-detection and disease severity. In addition, this single center study might not be representative of the entire Chinese pediatric population.

## Conclusions

In conclusion, we concluded that though *Mp* co-detection with viral and/or bacterial pathogens is common in clinical practice, there are no apparent differences between *Mp* mono-detection and *Mp* co-detections in terms of clinical features and disease severity. Our findings may eventually contribute to a better understanding of the implication of *Mp* co-detections in clinical practice, which is essential to improve preventive and therapeutic strategies.

## Data Availability

The datasets used and/or analyzed during the current study available from the corresponding author on reasonable request.

## Abbreviations

*09H1*: *Influenza A H1N1 pdm09*
*ADV*: *Adenovirus*
BALF: Bronchoalveolar lavage fluid
CAP: Community-acquired pneumonia
*Ch*: *Chlamydia*
CRP: C-reactive protein
*Flu A*: *Influenza A*
*Flu B*: *Influenza B*
*H3*: *Influenza H3N2*
*HBoV*: *Human bocavirus*
*HCoV*: *Human coronavirus*
*HMPV*: *Human metapneumovirus*
*HPIV*: *Human parainfluenza virus*
*HRV*: *Rhinovirus*
*Mp*: *Mycoplasma pneumoniae*
NPAs: Nasopharyngeal aspirates
PCR: Polymerase chain reaction
*RSV*: *Respiratory syncytial virus*
